# Buttress plate augmentation offers no clear clinical advantage over compression screws alone for Hoffa fractures: a systematic review and meta-analysis

**DOI:** 10.1007/s00590-026-05027-y

**Published:** 2026-11-14

**Authors:** Siddharth Khadilkar, Nishanth Muthusamy, Michael Sundahl, Ronak J. Mahatme, Shawn Moore, Anish Gangavaram, Michael J Beltran, Jonathan D Ellis

**Affiliations:** Department of Orthopaedic surgery and Sports Medicine, University of Cincinnati Medical Center, Cincinnati, USA

**Keywords:** Hoffa fracture, Buttress plate, Compression screw, Fracture fixation, Meta-analysis

## Abstract

**Purpose:**

To compare clinical, functional, and perioperative outcomes after fixation of Hoffa fractures using buttress plate augmentation with compression screws versus compression screws alone.

**Methods:**

PubMed, MEDLINE, Embase, and Scopus were searched from inception through 31 July 2026 for randomized or nonrandomized comparative studies of adults. Two reviewers independently screened studies, extracted data, and assessed risk of bias using ROBINS-I. Random-effects meta-analysis used restricted maximum likelihood estimation and Hartung–Knapp–Sidik–Jonkman confidence intervals. The protocol was registered in PROSPERO.

**Results:**

Four nonrandomized studies involving 143 patients (BPCS, 63; CS, 80) were included. BPCS was associated with greater knee range of motion at 4 months (mean difference [MD], 10.85°; 95% confidence interval [CI], 1.69–20.02; *p* = 0.04; I² = 0%) and longer operative time (MD, 23.03 min; 95% CI, 7.45–38.62; *p* = 0.02; I² = 84%). No significant differences were found in range of motion at ≥ 12 months, early or late functional knee scores, blood loss, or time to union. Overall risk of bias was moderate in one study and serious in three.

**Conclusion:**

Limited observational evidence suggests that buttress plate augmentation may improve early knee motion but prolongs operative time, without a demonstrated durable functional or union advantage. Because the evidence is sparse and at substantial risk of bias, fixation should be individualized according to fracture characteristics and intraoperative stability.

## Introduction

**Fig. 1:**
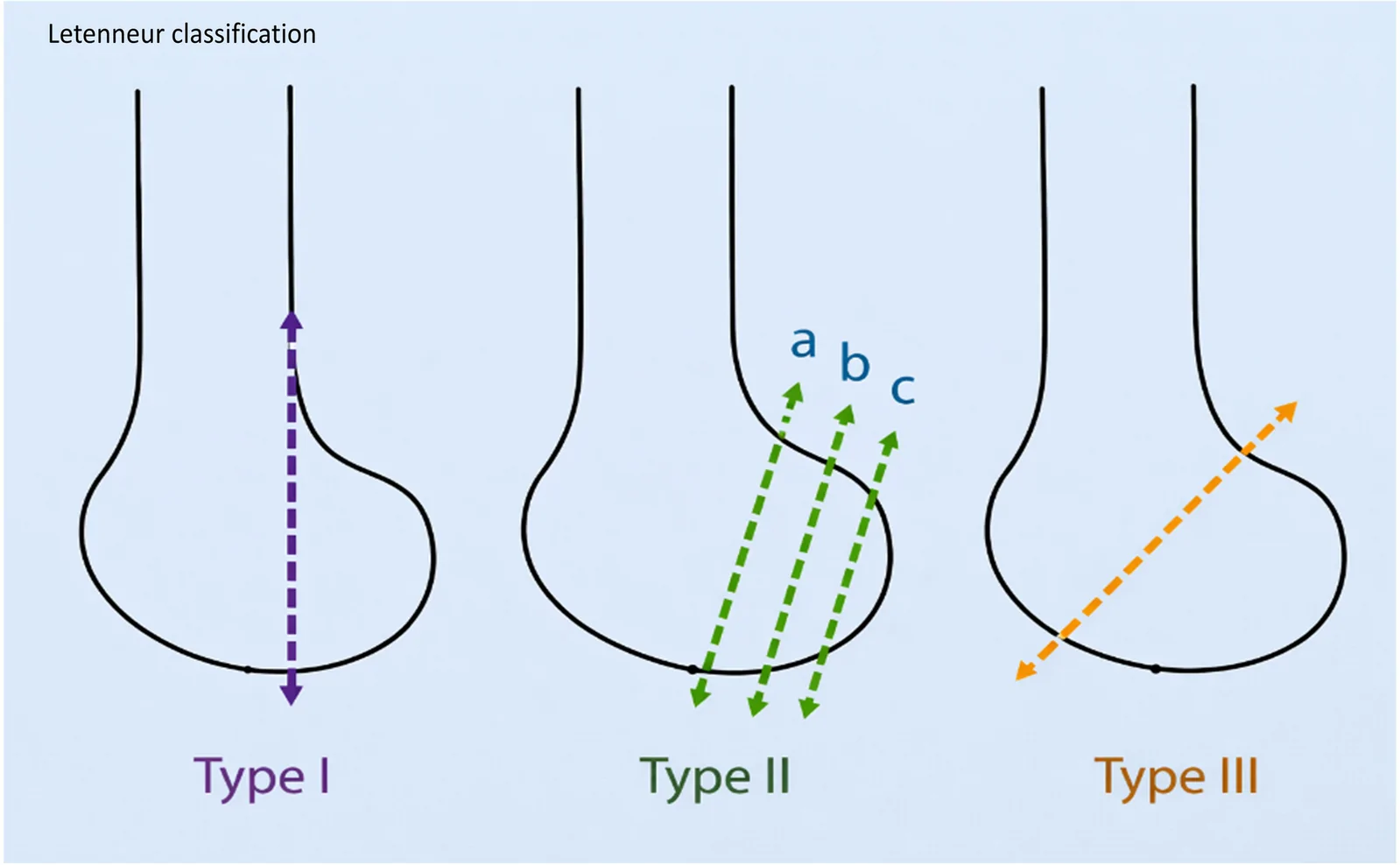
Letenneur classification of Hoffa fractures illustrating the orientation of coronal plane fracture lines involving the femoral condyles. Type I fractures are characterized by a vertical fracture line parallel to the posterior femoral cortex involving the entire condyle. Type II fractures demonstrate a fracture line located posterior to the attachment of the collateral ligaments and are subclassified into Type IIa, IIb, and IIc based on the superior-to-inferior location of the fracture fragment. Type III fractures are oblique fracture patterns extending anteriorly from the intercondylar notch toward the posterior condyle

Hoffa fractures are partial articular fractures of the posterior femoral condyle oriented in the coronal plane (AO/OTA 33-B3.2/3) [1]. These fractures are uncommon and account for 8.7–13% of all distal femoral fractures and 0.65% of all femoral fractures [2, 3]. These injuries were first described by Busch in 1869 and subsequently by Albert Hoffa in 1904 [4, 5]. Letenneur et al. subsequently classified these fractures into three principal types in 1978 (Fig. 1) [6].

Nonoperative treatment typically has been described for nondisplaced fractures but carries a risk of secondary displacement, nonunion, and knee dysfunction often needing delayed internal fixation and bone grafting [7–10]. The deforming force of the gastrocnemius muscle and shear forces across the posterior femoral condyle are the main causes of displacement. Operative fixation as a result is advised to achieve anatomical reduction, stability and allow for early mobilization [11–14].

Various fixation techniques like cancellous screws, headless screws, compression screws and buttress plating have been described in the literature [14–17]. Lag screws can provide interfragmentary compression with less extensive soft-tissue dissection, whereas buttress plates may improve resistance to shear and fragment displacement [18–21]. A recent systematic review evaluated surgical outcomes using broad, heterogeneous fixation categories that combine distinct constructs [22]. However, no previous meta-analysis has restricted inclusion to direct comparative studies comparing compression screws alone versus compression screws with buttress plate augmentation. The purpose of this systematic review and meta-analysis was to compare functional, radiographic, and perioperative outcomes of fixation of Hoffa fractures with isolated compression screw (CS) fixation versus compression screws plus additional buttress plate fixation (BPCS). It was hypothesized that plate augmentation does not improve outcomes.

## Materials and methods

### Search strategy

**Fig. 2:**
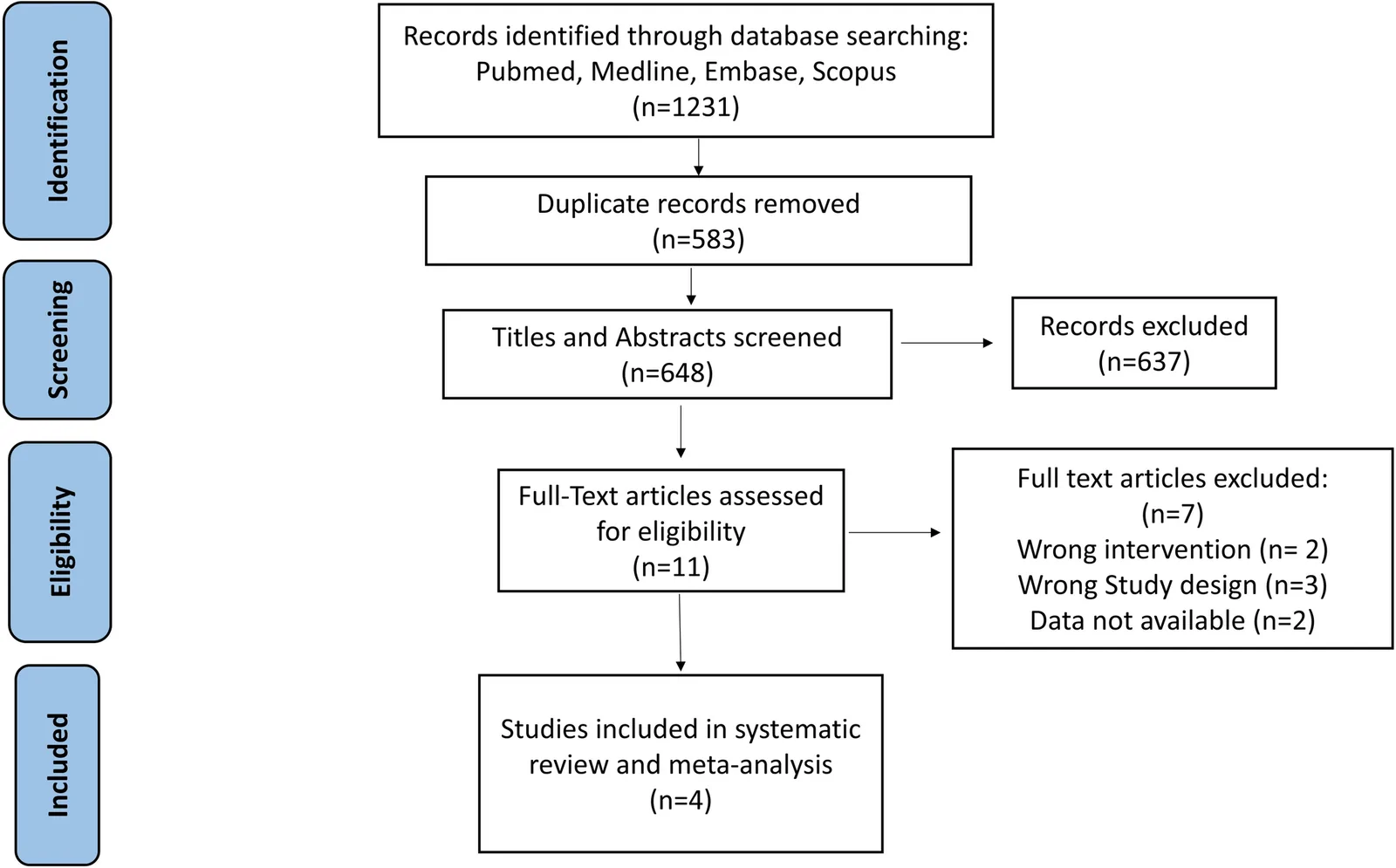
PRISMA flow diagram illustrating the study selection process for the systematic review and meta-analysis, including identification, screening, eligibility assessment, and final inclusion of studies

This systematic review and meta-analysis was conducted in accordance with the Cochrane Handbook for Systematic Reviews of Interventions and reported according to the Preferred Reporting Items for Systematic Reviews and Meta-Analyses 2020 statement and the Meta-analysis of Observational Studies in Epidemiology guidelines [23, 24]. PubMed, MEDLINE, Embase, and Scopus were searched from inception through July 31, 2026. Database-specific strategies combined terms related to Hoffa or coronal-plane distal femoral fractures, internal fixation, compression screws, and buttress plating. Search results were imported into Covidence (Veritas Health Innovation, Melbourne, Australia), where duplicate records were identified and removed. Two independent reviewers screened titles and abstracts, followed by full-text review of potentially eligible studies according to the predefined inclusion and exclusion criteria. Disagreements were resolved through discussion, with a third reviewer adjudicating unresolved discrepancies. Reference lists of included studies and relevant review articles were manually screened to identify additional eligible studies. The study selection process is illustrated in the PRISMA flow diagram (Fig. 2).

### Inclusion criteria

Studies were eligible if they: (1) included skeletally mature or adult patients with Hoffa fractures; (2) directly compared BPCS with CS fixation alone; (3) used a randomized or nonrandomized comparative design; and (4) reported extractable data for at least one prespecified outcome. Prespecified outcomes included knee ROM, functional knee scores, time to union, operative time, intraoperative blood loss, and complications.

### Exclusion criteria

Noncomparative case series, case reports, biomechanical studies, technical notes without comparative outcomes, reviews, editorials, conference abstracts without sufficient data, and duplicate reports were excluded. Reports for which full text or extractable comparative data could not be obtained after reasonable attempts were also excluded. The systematic review protocol was prospectively registered in PROSPERO (registration number blinded for peer review).

### Risk of bias assessment

Risk of bias was assessed using the 2016 version of the Risk of Bias In Non-randomized Studies-of Interventions tool (ROBINS-I). Two reviewers independently completed the ROBINS-I assessments. Disagreements were resolved through discussion and, when necessary, adjudication by a third reviewer. The following seven domains were evaluated: bias due to confounding, participant selection, intervention classification, deviations from intended interventions, missing data, outcome measurement, and selection of reported results. Each domain was graded as low, moderate, serious, or critical risk of bias, or no information.

Overall risk of bias was moderate in one study and serious in three, primarily because of confounding and participant selection; no study was judged to have a critical risk of bias (Table 1).

**Table 1:** Risk-of-Bias Assessment of Included Studies Using ROBINS-I

Study	D1: confounding	D2: selection	D3: classification of intervention	D4: deviation from interventions	D5: missing data	D6: measurement of outcome	D7: selection of reported result	Overall risk of bias
Hazarika [27]	Serious	Moderate	Moderate	Low	Low	Moderate	Moderate	Serious
Langen [21]	Serious	Moderate	Low	Moderate	Moderate	Moderate	Moderate	Serious
Vignesh [25]	Moderate	Low	Low	Low	Low	Moderate	Moderate	Moderate
Lu [28]	Serious	Moderate	Low	Low	Low	Moderate	Moderate	Serious

Domains: D1, bias due to confounding; D2, bias in selection of participants into the study; D3, bias in classification of interventions; D4, bias due to deviations from intended interventions; D5, bias due to missing data; D6, bias in measurement of outcomes; and D7, bias in selection of the reported result.

ROBINS-I, Risk Of Bias In Non-randomized Studies—of Interventions, 2016 version.

### Data extraction

Two reviewers independently extracted study design, setting, publication year, sample size, patient age and sex, fracture laterality, injury mechanism, Letenneur classification, fixation technique, follow-up duration, rehabilitation protocol, outcome definitions, measurement time points, means and standard deviations for continuous outcomes, and event counts for complications. Extracted data were cross-checked, and disagreements were resolved by consensus. The narrative summary and patient-level table in Vignesh et al. were internally inconsistent regarding group sizes and reported outcomes; therefore, group sizes, means, and standard deviations (SDs) were reconstructed from the patient-level Table [25]. The Neumann-Langen study included isolated screw fixation, isolated plate fixation, and combined plate-and-screw fixation. The isolated plate-only group was excluded because it did not meet the prespecified comparator definition [21]. When a study reported a mean and range without an SD, the SD was estimated using the method described by Wan et al. [26]

### Statistical analysis

Statistical analyses were performed using Review Manager Web (RevMan Web), version 11.4.0. A random-effects model was applied to account for clinical and methodological heterogeneity among the included studies. Mean differences (MDs) with 95% confidence intervals (CIs) were calculated for continuous outcomes reported in common units, including knee ROM in degrees, operative time in minutes, intraoperative blood loss in milliliters, and time to union in months. Functional outcomes were categorized as early follow-up at 3–4 months and later follow-up at ≥ 12 months. Standardized mean differences (SMDs) with 95% CIs were calculated for functional outcomes assessed using different validated scoring instruments; positive values favored buttress plate augmentation with compression screws. Between-study variance (τ²) was estimated using restricted maximum likelihood (REML), and 95% CIs for pooled effects were calculated using the Hartung–Knapp–Sidik–Jonkman (HKSJ) method. Statistical heterogeneity was assessed using τ² and the I² statistic. All statistical tests were 2-sided, and *p* < 0.05 was considered statistically significant. Leave-one-out sensitivity analyses were performed for outcomes reported by at least 3 studies by sequentially excluding each study and recalculating the pooled estimate.

## Results

### Study characteristics

Four nonrandomized comparative studies involving 143 patients met the eligibility criteria [21, 25, 27, 28]. The BPCS group had 63 (44.1%) patients and CS group had 80 (55.9%) patients. The mean age was similar between the BPCS and CS groups (40.45 ± 13.5 vs. 40.49 ± 14.1 years; *p* = 0.99), as was the proportion of female patients (47.6 vs. 36.3%; *p* = 0.17). No significant between-group differences were observed in laterality, mechanism of injury, or Letenneur fracture classification. Study and patient characteristics are summarized in Table 2.

**Table 2:** Characteristics of Included Studies and Baseline Patient Characteristics

Study	Study design	Comparison	No. of patients	Age, y	Sex, male/female, *n*	Laterality, left/right, *n*	Mechanism, RTA/fall, *n*	Letenneur type I/II/III, *n*	Follow-up, mo
Hazarika [27]	Non-RCT	BPCS vs. CS	10; 10	39 ± 9; 33.5 ± 7.3	7/3; 6/4	4/6; 7/3	7/3; 8/2	NR	NR
Neumann-Langen [21]	Non-RCT	BPCS vs. CS	14; 34	47.1 ± 24.2; 46.6 ± 17.3	6/8; 24/10	NR	NR	5/2/5^a^; 9/16/9	19.1 ± 24.1; 25.6 ± 29.7
Vignesh [25]	Non-RCT	BPCS vs. CS	15; 15	37.53 ± 8.58; 34.8 ± 9.25	5/10; 9/6	11/4; 4/11	9/6; 6/9	0/9/6; 9/6/0	NR
Lu [28]	Non-RCT	BPCS vs. CS	24; 21	39 ± 7; 38 ± 9.5	15/9; 12/9	10/14; 7/14	17/7; 15/6	8/6/10; 7/6/8	NR
Overall		BPCS vs. CS	63; 80	40.5 ± 13.5; 40.5 ± 14.1	33/30; 51/29	25/24; 18/28	33/16; 29/17	13/17/21; 25/28/17	
Overall unadjusted *P* value				0.99	0.17	0.24	0.66	0.13	-

For paired values, BPCS data are presented first, followed by CS data. Continuous variables are presented as mean ± SD, and categorical variables are presented as counts in the category order specified in the corresponding column heading. Overall age was calculated by combining study-level means, SDs, and sample sizes; for Vignesh et al., the group-specific SD was calculated from individual patient ages reported in the study. Overall categorical values represent available case totals and therefore may not equal the total treatment-group sample size because of incomplete reporting across studies. Overall unadjusted P values were calculated using the Welch independent-samples t test for age and Pearson chi-square tests for categorical variables.

Laterality and mechanism were available for 49 BPCS and 46 CS patients. Letenneur classification was available for 51 BPCS and 70 CS patients. Follow-up was not pooled because group-specific follow-up was reported by only one study.

BPCS, buttress plate plus compression screw; CS, compression screw alone; NR, not reported; RTA, road traffic accident; SD, standard deviation.

### Clinical outcomes

**Fig. 3:**
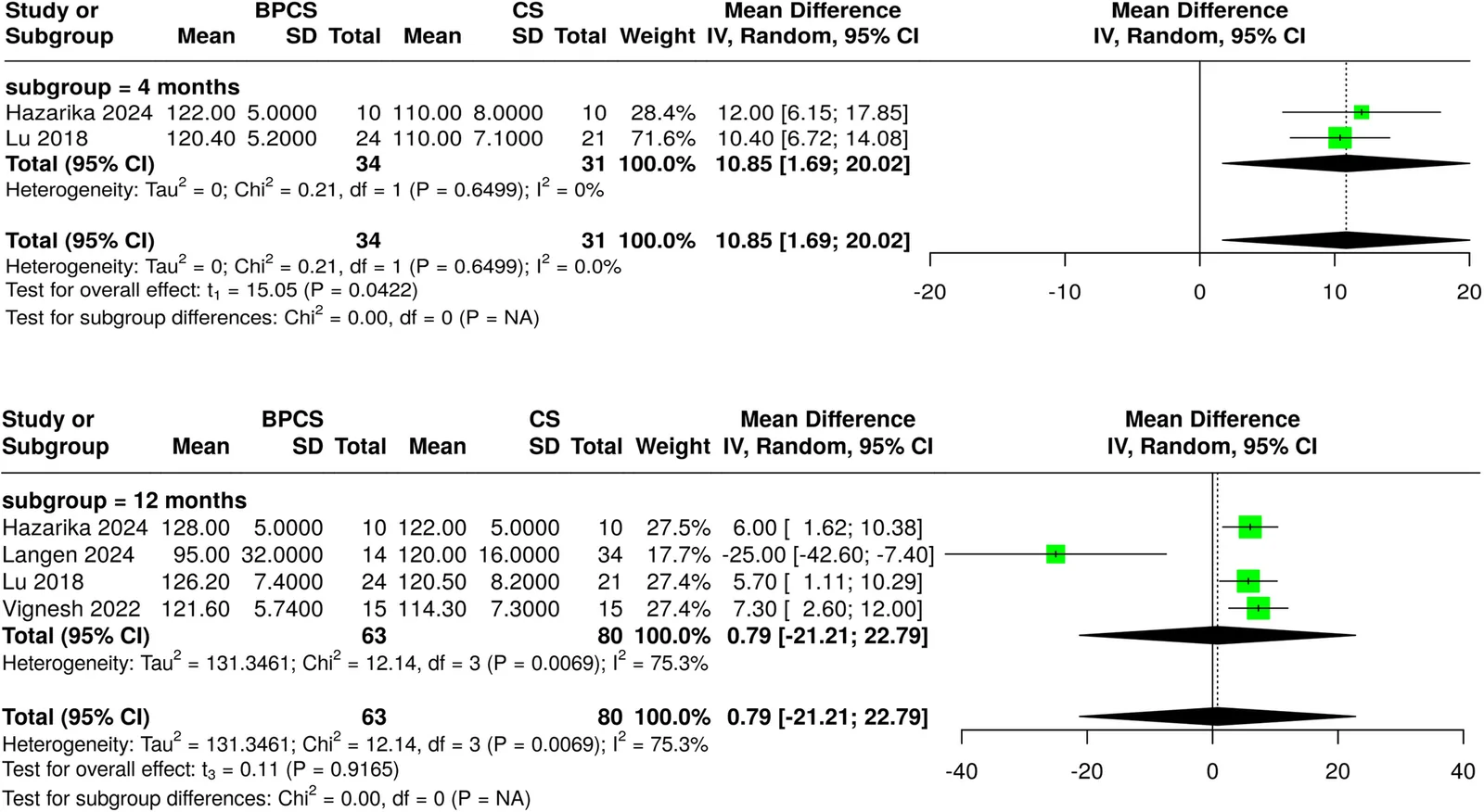
Forest plots comparing postoperative knee range of motion (ROM) between buttress plating with compression screw fixation (BPCS) and compression screw fixation alone (CS) at 4-month and ≥ 12-month follow-up intervals

**Fig. 4:**
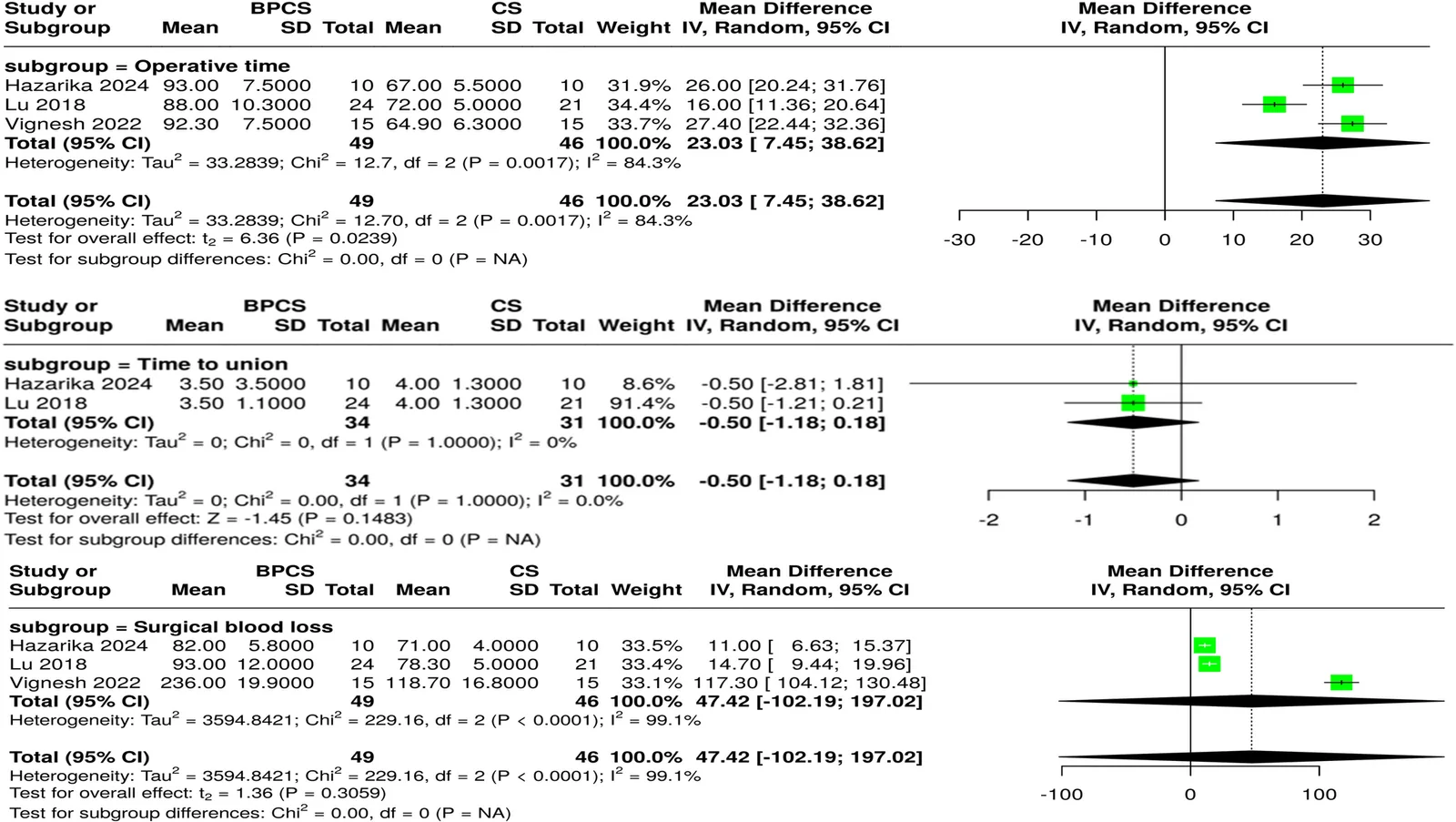
Forest plots comparing operative outcomes between buttress plating with compression screw fixation (BPCS) and compression screw fixation alone (CS), including operative time, time to union and surgical blood loss

**Fig. 5:**
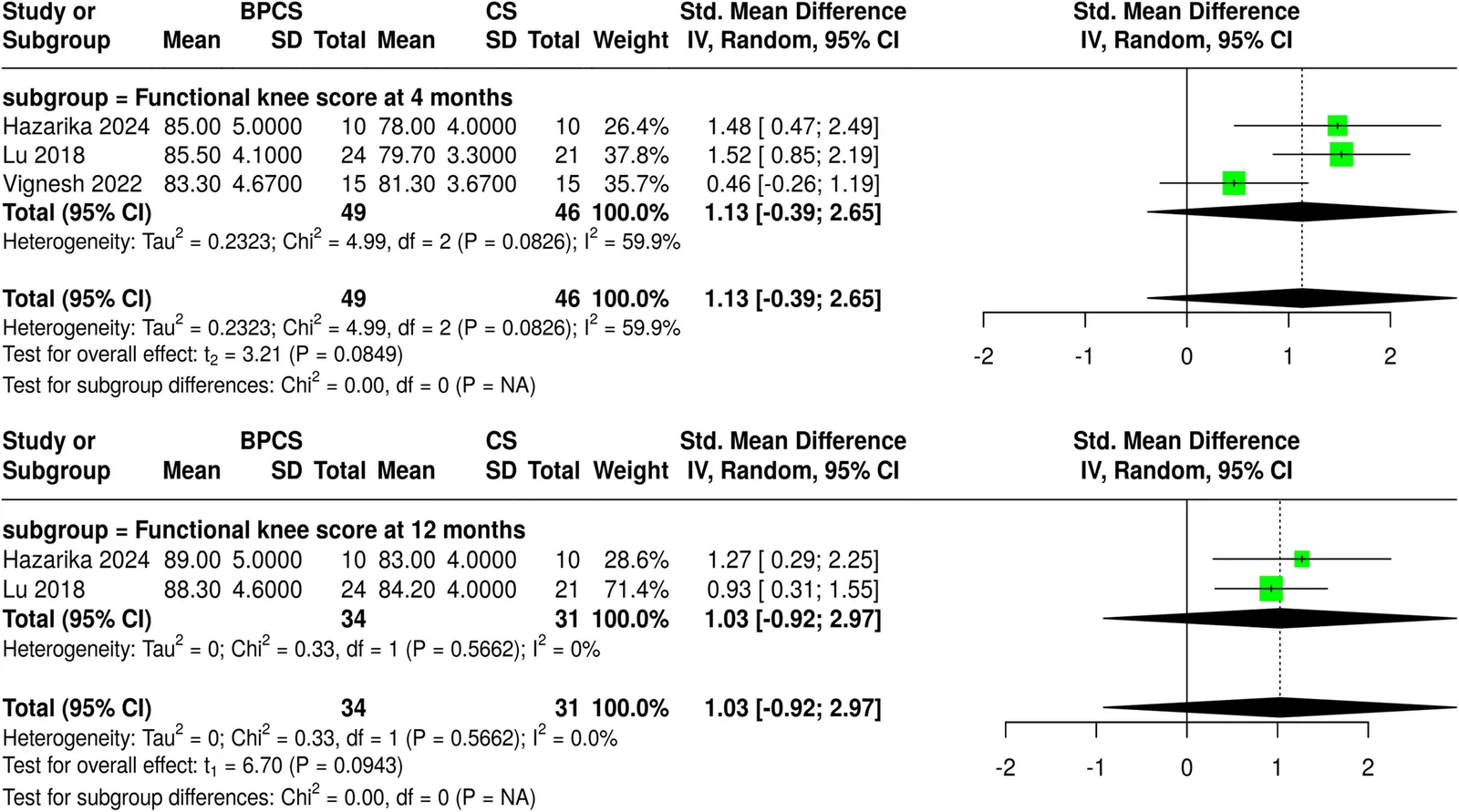
Forest plots comparing functional knee outcome scores between buttress plating with compression screw fixation (BPCS) and compression screw fixation alone (CS) at 4-month and ≥ 12-month follow-up intervals

Two studies reported knee ROM in 65 patients at 4 months [27, 28], while all four studies reported knee ROM in 143 patients at ≥ 12 months [21, 25, 27, 28]. Knee ROM was significantly greater in the BPCS group at 4 months compared with the CS group (MD, 10.85°; 95% CI, 1.69–20.02; *p* = 0.04; I² = 0%), however, there was no significant difference at ≥ 12 months (MD, 0.79°; 95% CI, − 21.21 to 22.79; *p* = 0.92; I² = 75.3%) (Fig. 3). Operative time was significantly longer in BPCS group compared with CS group (MD, 23.03 min; 95% CI, 7.45–38.62; *p* = 0.02; I² = 84.3%). There was no significant difference in average surgical blood loss between the BPCS and CS groups (MD, 47.42 mL; 95% CI, − 102.19 to 197.02; *p* = 0.31; I² = 99.1%) (Fig. 4). There was no significant difference in time to union between the BPCS and CS groups (MD, − 0.50 months; 95% CI, − 1.18 to 0.18; *p* = 0.15; I² = 0%) (Fig. 4). There was no significant difference in functional knee scores between BPCS and CS groups at 4 months (SMD, 1.13; 95% CI, − 0.39 to 2.65; *p* = 0.08; I² = 59.9%) and at ≥ 12 months (SMD, 1.03; 95% CI, − 0.92 to 2.97; *p* = 0.09; I² = 0%) respectively (Fig. 5).

### Sensitivity analyses

Leave-one-out analyses were performed for ROM at final follow-up, operative time, intraoperative blood loss, and early functional scores because these outcomes were reported by at least 3 studies. The direction of effect was unchanged after sequential exclusion of individual studies. For blood loss, exclusion of Vignesh et al. reduced I² from 99% to 18% while the pooled estimate remained statistically nonsignificant.

## Discussion

In this systematic review and meta-analysis of four comparative studies involving 143 patients with Hoffa fracture, buttress plate augmentation with compression screws (BPCS) was associated with 11 degrees greater knee ROM at 4 months but a 23-minute longer operative time compared to compression screws (CS) alone. The early ROM difference did not persist at ≥ 12 months, and no significant differences were observed in functional scores, blood loss, or time to union. Given the small samples and moderate-to-serious risk of bias, these findings do not establish superiority of either construct.

These findings complement but differ from those of Prakash et al., who evaluated 17 heterogeneous studies and group screw fixation and single-plate constructs together for comparison with dual plating [22]. By restricting inclusion to direct comparisons of compression screws with versus without buttress plate augmentation, the present analysis addresses a narrower clinical question and avoids pooling fundamentally different constructs. The greater specificity, however, resulted in a smaller evidence base and correspondingly imprecise estimates.

Hoffa fractures are exposed to substantial posterior shear forces, and biomechanical studies suggest that supplemental buttress plating can improve resistance to fragment displacement in selected fracture configurations [29–31]. Greater construct stability could explain the modest improvement in early ROM observed with BPCS, however, treatment allocation was not randomized, and plate augmentation was often selected for more unstable or comminuted fractures. The observed differences may therefore reflect fracture complexity, surgical approach, rehabilitation protocols, or surgeon preference rather than the fixation construct alone. Although several clinical series have reported satisfactory outcomes with both plate-augmented fixation and compression screws alone [32–35], comparative evidence remains limited to small retrospective and nonrandomized prospective studies, with no randomized controlled trial establishing the superiority of either fixation strategy [25, 28].

The absence of differences in final ROM, functional scores, and time to union suggests that both constructs may provide adequate fixation when appropriately selected and when anatomic reduction and stable fixation are achieved. Mean time to union was 3.5 months with BPCS and 4 months with CS, consistent with previously reported healing times [36–38]. The longer operative time associated with BPCS may reflect the additional exposure and dissection required for plate placement as well as greater fracture complexity in patients selected for plating. Accordingly, the clinical relevance of this difference likely depends on the fracture pattern and the patient’s ability to tolerate a longer procedure. Complications could not be meaningfully compared because reporting was inconsistent and was often not stratified by fixation techniques.

This study has several limitations. All included studies were small, nonrandomized observational studies subject to selection bias and confounding by indication. Considerable heterogeneity existed in fracture patterns, operative techniques, rehabilitation protocols, and outcome reporting, limiting statistical precision and generalizability. Larger prospective multicenter studies with standardized outcome reporting are needed. Until stronger evidence is available, fixation should be individualized according to fracture configuration, comminution, bone quality, surgical exposure, and intraoperative stability.

## Conclusion

For the treatment of Hoffa fractures, BPCS was associated with greater knee ROM at 4 months, but longer operative time compared with CS alone. No statistically significant differences were observed in ≥ 12-month ROM, functional knee scores, intraoperative blood loss, or time to union. The available evidence does not establish superiority or equivalence between these fixation techniques or support routine buttress plate augmentation for all Hoffa fractures. Fixation strategy should remain individualized according to fracture configuration, comminution, bone quality, intraoperative stability, surgical exposure, and patient-specific factors.

## Data Availability

All data analyzed in this systematic review and meta-analysis were obtained from the published studies cited in the manuscript. The extracted dataset used for the analyses is available from the corresponding author upon reasonable request.
